# Real-time forecasting of COVID-19 spread according to protective behavior and vaccination: autoregressive integrated moving average models

**DOI:** 10.1186/s12889-023-16419-8

**Published:** 2023-08-08

**Authors:** Chieh Cheng, Wei-Ming Jiang, Byron Fan, Yu-Chieh Cheng, Ya-Ting Hsu, Hsiao-Yu Wu, Hsiao-Han Chang, Hsiao-Hui Tsou

**Affiliations:** 1 Department of Life Science & Institute of Bioinformatics and Structural Biology, National Tsing Hua University, Hsinchu, Taiwan; 2 Institute of Population Health Sciences, National Health Research Institutes, 35 Keyan Road, Zhunan, Miaoli County 350 Taiwan; 3 Brown University, RI Providence, USA; 4 Graduate Institute of Biostatistics, College of Public Health, China Medical University, Taichung, Taiwan

**Keywords:** COVID-19, Forecasting, regARIMA, Vaccines, Nonpharmaceutical intervention

## Abstract

**Background:**

Mathematical and statistical models are used to predict trends in epidemic spread and determine the effectiveness of control measures. Automatic regressive integrated moving average (ARIMA) models are used for time-series forecasting, but only few models of the 2019 coronavirus disease (COVID-19) pandemic have incorporated protective behaviors or vaccination, known to be effective for pandemic control.

**Methods:**

To improve the accuracy of prediction, we applied newly developed ARIMA models with predictors (mask wearing, avoiding going out, and vaccination) to forecast weekly COVID-19 case growth rates in Canada, France, Italy, and Israel between January 2021 and March 2022. The open-source data was sourced from the YouGov survey and Our World in Data. Prediction performance was evaluated using the root mean square error (RMSE) and the corrected Akaike information criterion (AICc).

**Results:**

A model with mask wearing and vaccination variables performed best for the pandemic period in which the Alpha and Delta viral variants were predominant (before November 2021). A model using only past case growth rates as autoregressive predictors performed best for the Omicron period (after December 2021). The models suggested that protective behaviors and vaccination are associated with the reduction of COVID-19 case growth rates, with booster vaccine coverage playing a particularly vital role during the Omicron period. For example, each unit increase in mask wearing and avoiding going out significantly reduced the case growth rate during the Alpha/Delta period in Canada (–0.81 and –0.54, respectively; both *p* < 0.05). In the Omicron period, each unit increase in the number of booster doses resulted in a significant reduction of the case growth rate in Canada (–0.03), Israel (–0.12), Italy (–0.02), and France (–0.03); all *p* < 0.05.

**Conclusions:**

The key findings of this study are incorporating behavior and vaccination as predictors led to accurate predictions and highlighted their significant role in controlling the pandemic. These models are easily interpretable and can be embedded in a “real-time” schedule with weekly data updates. They can support timely decision making about policies to control dynamically changing epidemics.

**Supplementary Information:**

The online version contains supplementary material available at 10.1186/s12889-023-16419-8.

## Background

On March 11, 2020, the World Health Organization declared the global outbreak of 2019 coronavirus disease (COVID-19) to be a pandemic [1]. Over the course of the pandemic, variants of severe acute respiratory syndrome coronavirus 2 (SARS-CoV-2) have caused waves of COVID-19 cases all over the world, posing significant healthcare, economic, and social challenges. As of March 31, 2022, SARS-CoV-2 infection was reported to have been detected in 529.97 million individuals and to have caused the deaths of 6.32 million individuals [2].

Governments have attempted to control the spread of SARS-CoV-2 infection by mandating and promoting protective behavior [3]. Facemask use, stay-at-home orders, social distancing, and lockdowns were mandated in Italy [4], France [5], Canada [6], and Israel [7], the countries of interest in the present work. Such protective behaviors have been shown to control the COVID-19 pandemic, before and after the administration of vaccines for all SARS-CoV-2 variants [8, 9]. For example, mask wearing combined with social distancing effectively flattened the epidemic curve [10]. Vaccine administration has been documented to be integral to stopping epidemic spread, and the safety and efficacy of current vaccines have been proven [11–15].

Mathematical and statistical models enabling the real-time forecasting of the paths of COVID-19 epidemics and estimation of the impacts of protective behaviors, including vaccination, have also been key for outbreak control. Such forecasting is an essential part of public health policy, guiding the timely implementation of vector control operations and mitigating outbreak risks. It has been used successfully during pandemics/epidemics of influenza (H1N1-2009) [16], dengue fever [17, 18], and Ebola [19].

Several mathematical methods and machine learning models are applied to forecast future epidemic trends [20] and explore the COVID-19 transmission factors. The first approach is the regression model. Bo Y et al. [3] used the generalized linear mixed model to estimate the effectiveness of non-pharmaceutical interventions (NPIs) for containing COVID-19. Amuedo-Dorantes C et al. [21] examined the impact of NPIs on COVID-19 mortality rates using linear regression. Rustagi V et al. [22] performed linear regression to analyze the effects of vaccinations. Alshogran OY et al. [23] used univariable and multivariable regression to indicate that the COVID-19 fatality rate exhibited a positive correlation with the percentage of individuals aged over 60 years. However, previous studies appear to have not aligned with the assumption of independent errors required for regression analysis, which indicates that the regression model is not very suitable for time series data.

The second approach is machine learning. Several models have been developed for forecasting purposes. Yeşilkanat CM [24] used a random forest machine learning algorithm to forecast global daily cases. Ballı S [25] indicated that the support vector machine method performed well in analyzing the temporal patterns of cumulative COVID-19 data. Kumar Y et al. [26] predicted ten countries' pandemics by deep learning. Shetty RP et al. [27] used an artificial neural network to predict COVID-19 cases in one state of India. Chimmula VKR et al. [28] used long short-term memory forecast Canada transmission. Though machine learning is a popular technique, there are some weaknesses. For example, being a “block box model” lacks interpretation [29] and demands substantial volumes of data [20].

The third approach is time series forecasting models, such as autoregressive model, moving average model, autoregressive moving average model, and autoregressive integrated moving average (ARIMA) model. These models ascertain the stationary condition of the data. Alzahrani SI et al. [30] indicated ARIMA model outperformed other time series forecasting models. The ARIMA model can explain the complex autocorrelation found in datasets and be applied quickly in a wide variety of fields, including economics [31], manufacturing [32], and public health [33]. It has been used recently to predict the spread of COVID-19 in many countries [34–36]. Modified ARIMA models have been developed, with current examples including a hybrid ARIMA–wavelet-based forecasting model [37], a dynamic hybrid with a modified susceptible–exposed–infected–recovered–dead model [38], a hybrid ARIMA–discrete wavelet decomposition model [39], and a regression model with ARIMA errors (regARIMA) [40, 41] which have been applied to forecast pandemic trends and interpret additional factors as external regressors.

A review of the ARIMA model literature through February 4, 2023, suggests that only human mobility has been used to predict COVID-19 transmission before universal vaccination implementation in such models [42–46], and that vaccination was included as a predictor in only one study [47]. With increasing vaccination coverage and SARS-CoV-2 evolution, a comprehensive set of factors should be examined and vaccination and other protective behaviors should be included as predictors to improve dynamic forecasting.

With the objective of capturing the real-time dynamics of the pandemic and promptly addressing the associated challenges, we aimed to develop an innovative ARIMA model designed to provide accurate predictions while also effectively identify the critical determinants. In this longitudinal study, we used newly developed real-time ARIMA models to improve the accuracy of COVID-19 trend prediction, with the inclusion of vaccine coverage and protective behaviors as adjustment and predictive variables, respectively. We compared predictor effects on outbreak progression in the pre- and post-vaccine phases in four countries. Our models can help members of the public, policymakers, and healthcare professionals gain a deeper understanding of the COVID-19 pandemic, assess trends in the spread of viruses in real time, and determine the effectiveness of outbreak control strategies.

## Method

### Study areas

This study was conducted with data from France, Italy, Canada, and Israel. The first three countries were selected due to the similarity of their socioeconomic characteristics [i.e., real gross domestic product per capita] [48] and high baseline COVID-19 risk level (based on the obesity prevalence, proportion of the population aged > 65 years, and international arrivals) [49, 50]. These countries also experienced a significant first wave of COVID-19 in the first half of 2020, followed by waves in the Alpha & Delta (January 1–November 25, 2021 and Omicron (November 26, 2021–March 29, 2022) periods [2]. Moreover, these countries have different types of government that implemented various policy-driven responses, enabling the examination of a broad scope of COVID-19 control strategies [33]. Israel was chosen because of its leading vaccination promotion and implementation efficiency [51].

### Research design

**Fig. 1:**
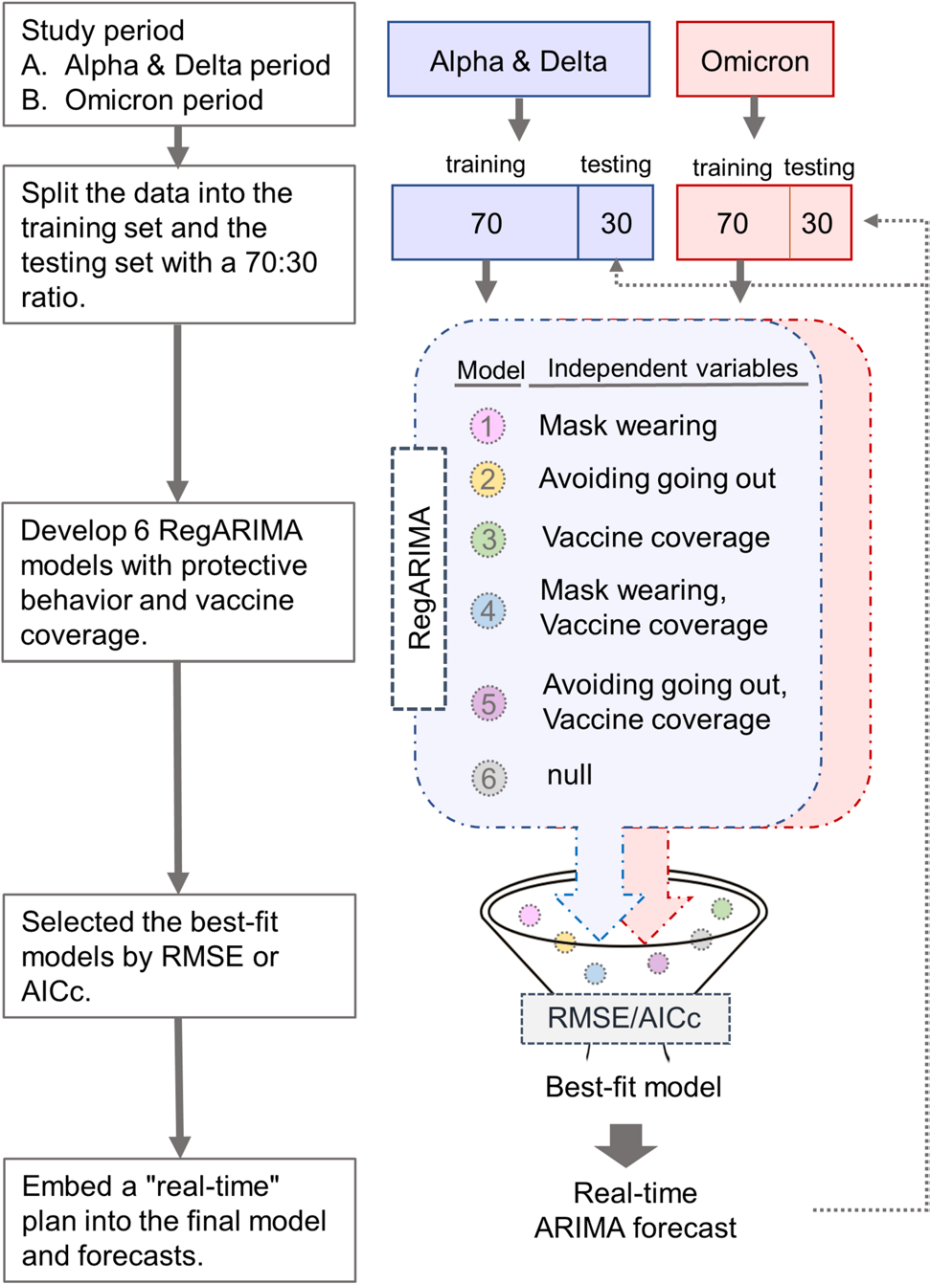
Flowchart of Reg-ARIMA model development for real-time COVID-19 forecasting

Figure 1 shows the research design. We used three primary datasets: epidemiological variables, protective behavior, and vaccine coverage. The epidemiological variables [COVID-19 case growth rate, number of intensive care unit (ICU) patients with COVID-19 per million, and number of deaths due to COVID-19 per million] served as dependent variables. Protective behaviors (mask wearing and avoiding going out) and vaccine coverage (proportions of the total national population vaccinated fully and having received booster doses) served as independent variables. We examined and compared the Alpha & Delta and Omicron periods.

In most ARIMA studies, large proportions of data are allocated to training and model building. Data series are categorized as in-sample and out-of-sample, at ratios such as 70:30 [52, 53], 80:20 [54], and 90:10 [55]. In this study, we allocated 70% of the data for training and 30% for testing. We also categorized data as in-sample and out-of-sample at ratios of 80:20 and 90:10 for sensitivity analyses to evaluate the robustness of our models in different ratios. We developed six regARIMA models with lags on protective behaviors and vaccine coverage, which profoundly affected the development of the dependent epidemiological variables [56]. The best-fitting models for each period were selected according to the root mean square error (RMSE) and corrected Akaike information criterion (AICc) and applied for the real-time forecasting of COVID-19 outbreaks and conditions.

### Data sources

The period of analysis was January 2021–March 2022. We retrieved epidemiological and vaccine coverage data from Our World in Data [2]. The database is public, updated in real time, and contains epidemic information collected from governments around the world and news agencies. The data on protective behaviors from the Imperial College of London’s YouGov COVID-19 Behavior Tracker Data Hub [57], which is a global, anonymized, longitudinal, and weekly web-based survey. YouGov ensures the data is representative through randomly selected and stratified samples. The small deviations were corrected by post-stratification weights [58]. YouGov data has been extensively utilized in numerous studies to capture perceptions and responses of individuals toward COVID-19 [59–62]. The utilization of weekly data is justified by multiple factors. Firstly, it enables the incorporation of the weekend effect and reduces the need for excessive adjustments to COVID-19 case numbers, making it a preferable option compared to daily data [63]. Secondly, the survey data is updated weekly, ensuring consistency in the chosen time unit. Third, given that many government policies are made on a weekly basis [64]. Taking these factors into account, emphasizing a weekly frequency becomes crucial for effective forecasting.

### Dependent variables

The COVID-19 case growth rate, rather than the number of new cases per million, was selected because it better reflects epidemic trends and meets the stationary condition for time-dependent trends, thereby enabling more accurate prediction [42, 43]. Furthermore, the variance of the series is stabilized by this taking of a logarithmic approach [63]. We calculated the log weekly case growth rate (*Y*_*t*_) using the method of Karaivanov et al. [6]:

$${Y}_{t}=\Delta \mathit{ln}\left({C}_{t}\right)=ln{ C}_{t}- ln{ C}_{t-1},$$

**Fig. 2:**
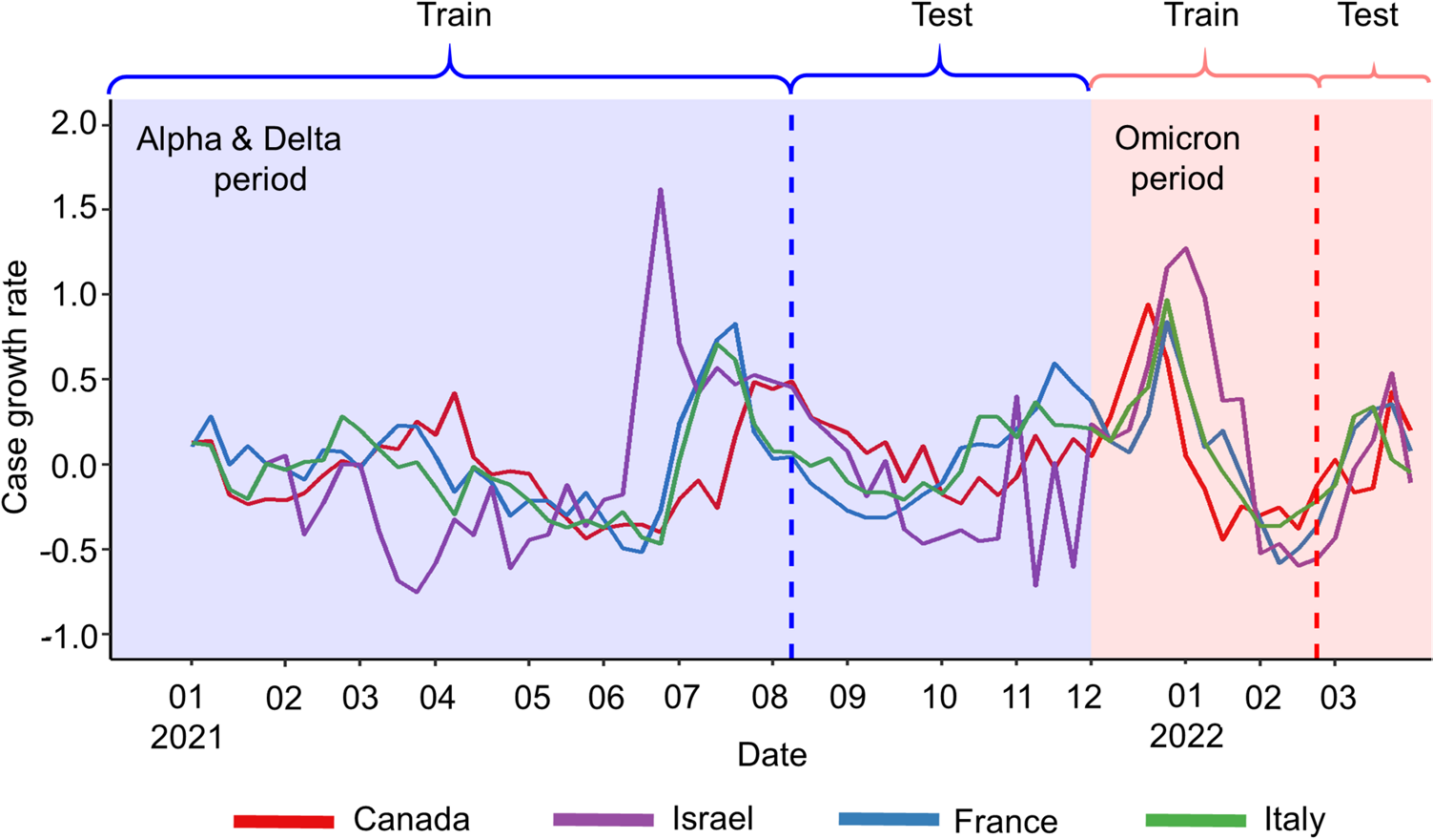
Weekly case growth rates. The Alpha & Delta (blue-shaded area) and the Omicron period (pink-shaded area) started from January 2021 to November 2021 and December 2021 to March 2022, respectively. Train-test ratio was 70:30, split by dash line

Where ${C}_{t}$ is the number of cases in week *t*; ${C}_{t-1}$ is the number of cases in the last week. The case growth rates in the four countries are shown in Fig. 2.

The two other variables are the weekly numbers of ICU patients with COVID-19 and deaths due to COVID-19 per million which were calculated as the sum of the 7-day numbers of ICU patients and deaths, respectively.

### Protective behaviors

Protective behaviors were measured by asking the question “Thinking about the last 7 days, how often have you taken the following measures to protect yourself or others from coronavirus (COVID-19)? As a reminder, please exclude any measures that you have already taken for reasons other than coronavirus (COVID-19)” [65]. For mask wearing and avoiding going out, we used the response statements “Worn a face mask outside your home (e.g., when on public transport, going to a supermarket, going to the main road)” and “Avoided going out in general,” respectively. Possible responses were “Always,” “Frequently,” “Sometimes,” “Rarely,” and “Not at all,” which we back-coded so that higher scores reflected greater adherence to protective behavior policies [66], taking the values of 4, 3, 2, 1, and 0, respectively. We calculated 7-day averages to obtain weekly data.

### Vaccine coverage

The proportion of the population vaccinated fully, defined following Tsou et al. [50] as the receipt of two vaccine doses, was calculated as:

$${V}_{t}=\frac{{V{\prime}}_{t}}{{2P}_{t}},$$

Where ${V{\prime}}_{t}$ is the total number of doses that the country administered and ${P}_{t}$ is the country's total population in week *t*.

The booster dose variable was used for adjustment during the Omicron period due to the flattening of the cumulative fully vaccinated population curve (S1 Fig.) [67]. It was defined as the ratio of the total number of booster COVID-19 vaccine doses administered per 100 people to the country’s total population.

### regARIMA model

The ARIMA (p,d,q) mathematical model was developed by Box and Jenkins. The autoregressive (AR) part uses past values to predict future values. The integration (I) uses the difference to make the time series stationarity. The moving average (MA) part sums the past error value. The p, d, and q are the orders of the autoregressive, integrated, and moving average. There are some steps for model selection. First, Box-Cox transformation is used to stabilize the variance. Second, the unit-root test is used to sure the time series stationarity. Third, autocorrelation function (ACF) and partial autocorrelation function (PACF) are used to select the order. Then, try the models we select and use AICc and RMSE to choose the best-performed model. Last, check the residuals are white noise [68].

The regARIMA time-series analysis model used in this study combines the benefits of ARIMA and linear regression, capturing autocorrelations in the data and enabling the inclusion of exogenous variables to improve forecast performance [40]. Additionally, its output is easier to interpret than is that of ARIMA models with explanatory variables (ARIMAX), which belong to the same hybrid model family [68], and regARIMA model use has been proposed for complex research objects [69]. Thus, regARIMA is the most appropriate model for our study.

In models 1–3, mask wearing, avoiding going out, and vaccine coverage, respectively, served as independent variables. Models 4 and 5 were adjusted for vaccine coverage and included mask wearing and avoiding going out, respectively. Model 6 was a base model developed for comparison. The model formulae were:

**Table:** 

	model	
$${Y}_{t}=C+{n }_{t}+\left\{\begin{array}{c}\begin{array}{c}{\beta }_{1}{M}_{t-l} \\ {{\beta }_{1}G}_{t-l} \\ {{\beta }_{1}V}_{\left(f,b\right)t-l} \end{array}\\ \begin{array}{c}{{\beta }_{1}M}_{t-l}+{{\beta }_{2}V}_{\left(f,b\right)t-l} \\ {{\beta }_{1}G}_{t-l}+{{\beta }_{2}V}_{\left(f,b\right)t-l} \\ \end{array}\end{array}\right.$$	(1) (2) (3) (4) (5)	,

$${n}_{t}={\varphi }_{1}{n}_{t-1}+\cdots \cdots +{\varphi }_{p}{n}_{t-p}+{\theta }_{1}{\varepsilon }_{t-1}+\cdots \cdots +{\theta }_{q}{\varepsilon }_{t-q}+{\varepsilon }_{t},$$

Where* C* is a constant, ${n}_{t}$ represents ARIMA errors, $\beta is$ estimates of the coefficient, and ${M}_{t-l}$, ${G}_{t-l}$, ${V}_{\left(f\right)t-l}$, and ${V}_{\left(b\right)t-l}$ represent mask wearing, avoiding going out, full vaccination, and booster doses in $t-l$ week, respectively, ( *l* is the time lag, *l* = 2 for the case growth rate and number of ICU patients per million and *l* = 4 for the number of deaths per million) [6, 70]. When incorporating the predictors (protective behavior and vaccination data), a comprehensive literature review is conducted as a preliminary step to understand the time lag between the predictors and the outcomes. The notation ${\varphi }_{i}$ is the parameter of autoregression for *i* = 1, …, *p*, where *p* is the number of lag observations in the model, also known as the lag order. Moreover, ${\theta }_{j}$ is the parameter of moving average for *j* = 1, …, *q*, where *q* is the order of the moving average. The notation ${\varepsilon }_{t}$ is a random error or residual term for the $t$ th week. Models 1 to 5 listed in Eq. (1) – (5) are regARIMA, while model 6 is an ARIMA model that estimates values using autoregressive without any predictors.

### Evaluation of model performance

We used the RMSE as a measure of the model’s predictive performance in the Alpha & Delta period, as in several previous studies [17, 55, 71, 72]:

$$\mathrm{RMSE}=\sqrt{\frac{1}{n}\sum_{t=1}^{n}{[{Y}_{t}-\widehat{{Y}_{t}}]}^{2}},$$

where ${Y}_{t}$ is the actual number of dependent variables at time *t*, $\widehat{{Y}_{t}}$ is the forecast dependent variable value, and* n* is the sample size. Lower values reflect more accurate forecasting.

We used the AICc, recommended for the analysis of short time series [40], to evaluate model performance in the Omicron period because the sample size was < 20. The AICc was defined as:

$$AICc=-2\mathrm{log}\left(L\right)+2k+\frac{2k\left(k+1\right)}{n-k-1},$$

Where* L* is the likelihood function, *k* is the total number of parameters, and* n* is the sample size. Lower values reflect better model performance.

### Real-time forecasting of the case growth rate

For real-time forecasting with the best-fitting model, the data were updated every 1–2 weeks and the model was refit with the newly updated dataset. The model was run using the auto.arima function of the "forecast" package in R 4.0.2 [73].

## Results

### Model estimates

**Fig. 3:**
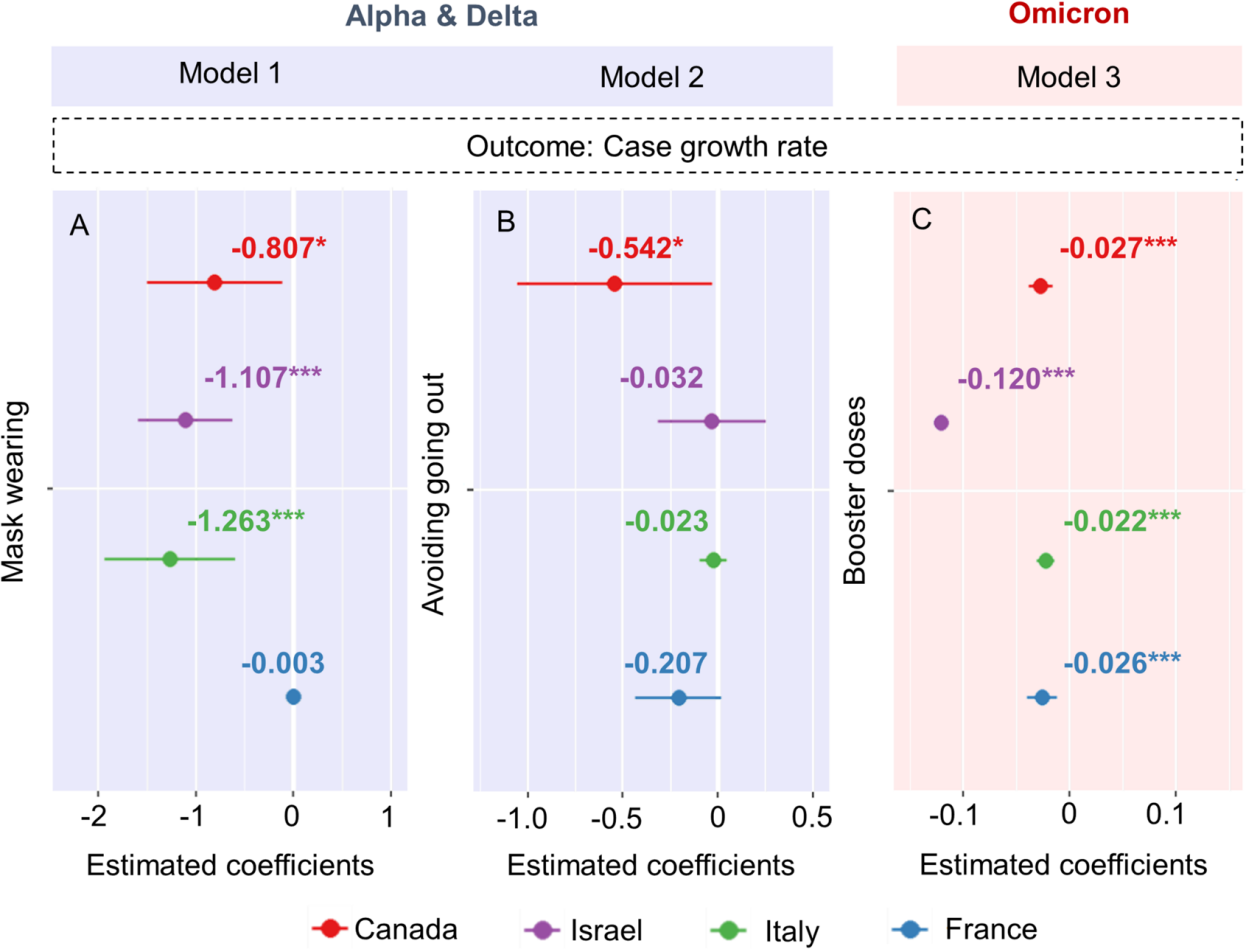
Relationships between predictors and the case growth rate in the Alpha & Delta (A and B for Model 1 and Model 2, respectively) and Omicron (C for Model 3) periods. *p < 0.05, **p < 0.01, ***p < 0.001

Figure 3A and B displays the relationship between the case growth rates and protective behaviors during the Alpha & Delta period. The estimates produced by all models with and without adjustment for vaccine coverage are provided in S1 Table. S1 Table also shows the p, d, and q obtained from ARIMA models. All coefficient for the protective behaviors were negative, indicating that the case growth rate decreased with adherence to protective behavior policies. For example, each unit increase in mask wearing and avoiding going out significantly reduced the case growth rate in Canada (–0.807 and –0.542, respectively; both *p* < 0.05; Fig. 3A). It is equivalent to a 2.24- and 1.72- fold reduction in the number of infected people compared to the original number during that week, respectively. In the Omicron period, each unit increase in the number of booster doses resulted in a significant reduction of the case growth rate in Canada (–0.027), Israel (–0.120), Italy (–0.022), and France (–0.026; all *p* < 0.05; Fig. 3C, S2 Table). It is equivalent to a 1.03-, 1.13-, 1.02-, and 1.03-fold reduction in the number of infected people compared to the original number during that week, respectively. S2 Table also shows the orders of p, d, and q obtained from ARIMA models.

**Fig. 4:**
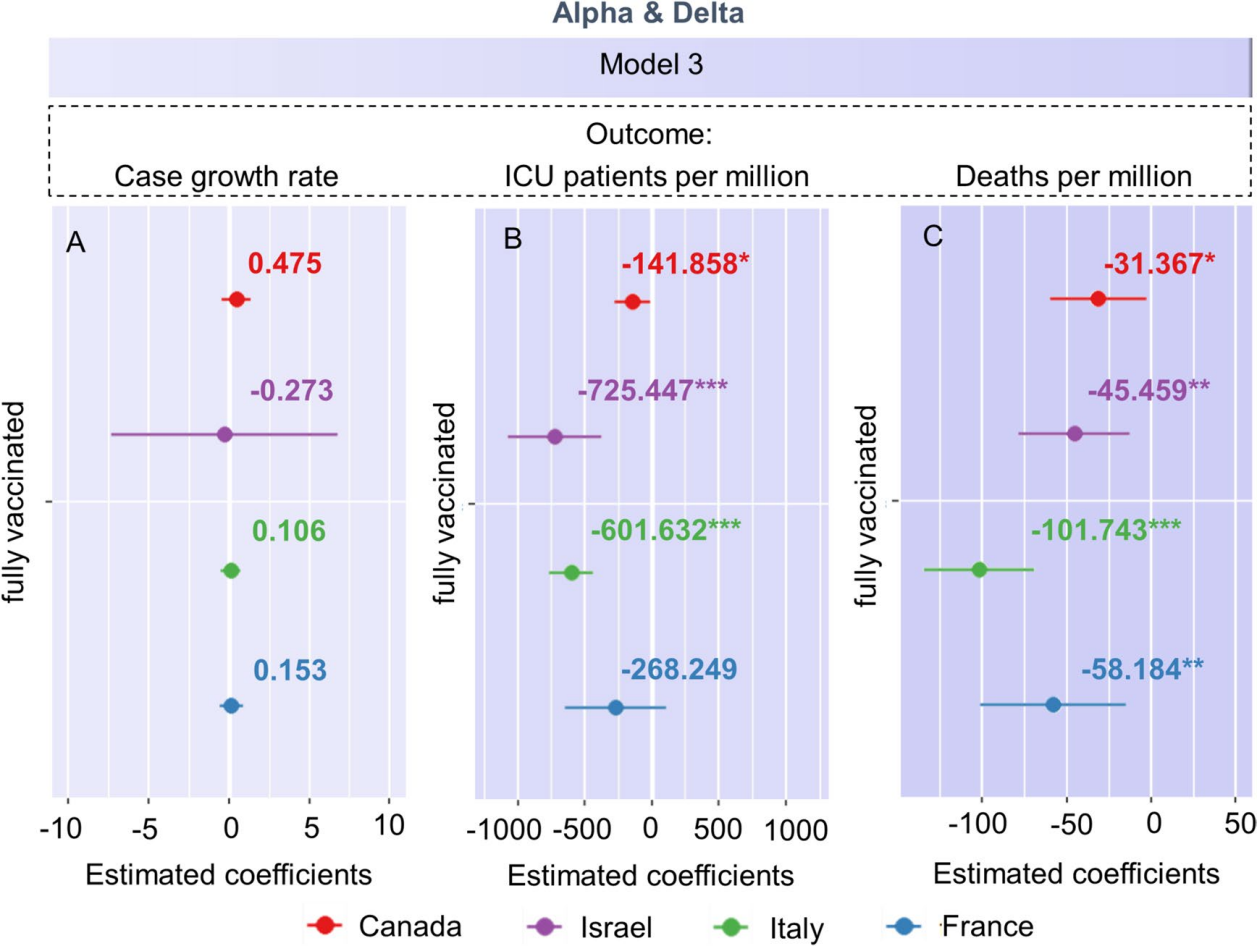
Effects of full vaccination. Effects of full vaccination on the (**A**) case growth rate, (**B**) number of ICU patients with COVID-19 per million, and (**C**) number of deaths due to COVID-19 per million in the Alpha & Delta period. *p < 0.05, **p < 0.01, ***p < 0.001

Figure 4 shows the effects of full vaccination on the epidemiological variables in the Alpha & Delta period. Full vaccination did not reduce the weekly case growth rate, but significantly reduced the numbers of ICU patients with COVID-19 and deaths due to COVID-19 per million in most countries. S1 Fig. shows the effects of full vaccination on the epidemiological variables in the Omicron period.

### Model selection

Model performance is quantified in Table 1. RMSE values for the Alpha & Delta period in almost all of the countries were lowest with model 4, indicating that this model had the best fit. According to this model, which was adjusted for full vaccination, each point increase in mask wearing was associated with a reduction in the case growth rate in Italy (–1.268, *p* < 0.05), Israel (–1.101, *p* < 0.05), Canada (–0.701, *p* > 0.05), and France (–0.020, *p* > 0.05). For the Omicron period, AICc values were lowest with model 6, which was thus designated the best-fitting model.

**Table 1:** Predictive performance of the regARIMA models

Outcome: weekly case growth rate							
period	country	Model 1	Model 2	Model 3	Model 4	Model 5	Model 6
Alpha & Delta	**Canada**						
	RMSE	0.1369	0.1400	0.1447	0.1351	0.1418	0.1479
	**Israel**						
	RMSE	0.2688	0.3448	0.3613	0.2687	0.3437	0.3614
	**Italy**						
	RMSE	0.1391	0.1520	0.1527	0.1391	0.1498	0.1530
	**France**						
	RMSE	0.1646	0.1583	0.1643	0.1635	0.1624	0.1647
Omicron	**Canada**						
	AICc	12.26	8.07	11.04	10.47	12.71	5.55
	**Israel**						
	AICc	15.14	21.19	18.51	18.59	19.99	13.66
	**Italy**						
	AICc	3.09	5.99	8.06	7.71	9.6	4.68
	**France**						
	AICc	9.5	9.13	13.08	12.67	13.57	6.35

*RMSE* Root mean square error

*AICc* Corrected Akaike information criterion

The results were obtained with the data split at a 70:30 ratio. Similar results were obtained with an 80:20 and a 90:10 split (S3 – S6 Table).

### Real-time forecasts

**Fig. 5:**
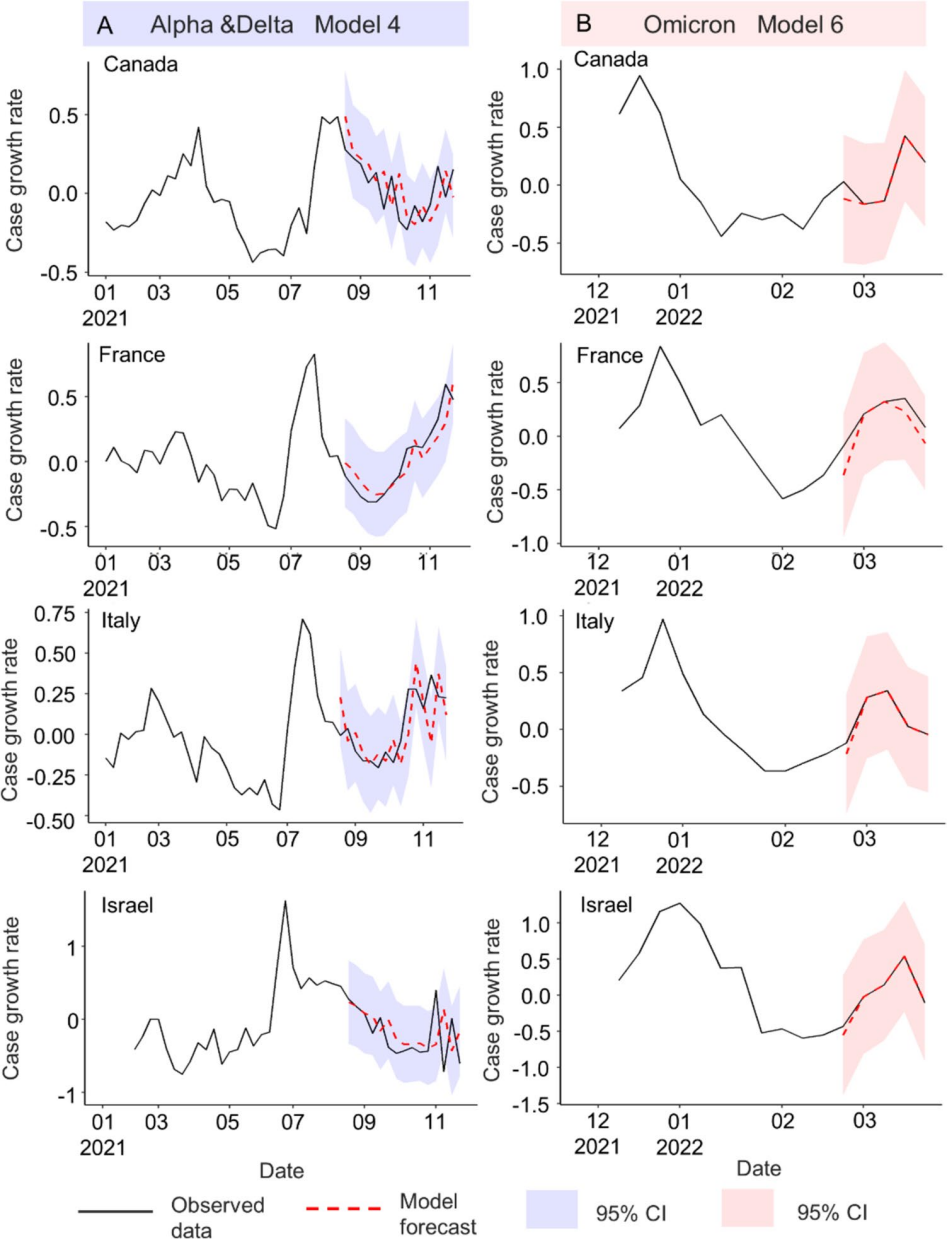
Real-time forecasts of the case growth rate in the Alpha & Delta (**A**) and Omicron (**B**) periods

The results of real-time forecasting of the case growth rates in the Alpha & Delta and Omicron periods using models 4 and 6, respectively, are shown in Fig. 5. The starting dates for prediction (first dates in the testing sets) were November 29, 2021 and February 28, 2022, respectively. The forecast obtained with model 4 was slightly delayed, whereas that obtained with model 6 was synchronized with the observed data. Overall, however, the models enabled accurate prediction.

## Discussion

In this study, we developed and tested novel regARIMA models that incorporate self-reported protective behaviors and vaccine coverage for the forecasting of COVID-19 trends. The models showed good accuracy and can play important roles in the prevention of epidemic spread.

During the Alpha & Delta period of the COVID-19 pandemic, our models 1 and 2 capture the importance of mask wearing and avoiding going out during this period, which both reduce the case growth rate. The results can be supported by other research. Chu et al. [74] indicated that face mask use can reduce the infection risk by a systematic meta-analysis. McGrail et al. [75] find that the government implementing social distancing policies can reduce the COVID-19 spread rate through the statistical modelling. Furthermore, the risks of hospitalization and mortality due to COVID-19 were more severe than those of seasonal influenza [76, 77], thus increasing people's willingness to engage in protective behavior. The high protective behavior frequency also makes us capture the effect of mask wearing and avoiding going out.

The model suggests that vaccination coverage has a limited impact on the case growth rate. We believe that vaccine hesitancy plays a significant role in the selected countries. For example, a global study indicates Italy and France’s vaccine acceptance rates are lower than 60% [78]. However, full vaccination significantly reduced the number of ICU patients and deaths attributable to COVID-19, in agreement with previous findings [79–81]. Bajema et al. [79] studied the effectiveness of the vaccine, they reported that the vaccine effectiveness of prevent COVID-19 hospitalization was 80% and 95% in adults aged ≥65 and 18-64 respectively. Victora et al. [81] investigated the vaccine on the association of deaths. The results indicated that vaccination significantly declines mortality in elderly people.

In the Omicron period, we observed that high booster dose rates significantly reduced the case growth rate. These results may be attributable in part to governments’ strong promotion of vaccine boosters due to the highly infectious nature of the Omicron variant early in this period. Full vaccination coverage in the countries of interest in this study exceeded 90% in January 2022.

In contrast to the Alpha & Delta period, it was observed that the efficacy of mask-wearing and adherence to stay-at-home orders in containing the pandemic has dwindled during the Omicron stage. This study conjectures that some reasons may cause the result. First, the Omicron variant of SARS-CoV-2 is more infectious than the Delta variant but is associated with lower hospitalization and mortality rates [82]. Second, according to the risk compensation hypothesis, the protective behaviors will decrease in vaccinated individuals. For example, Liang et al. [83] find that population mobility increases as vaccine coverage increases. In light of the factors mentioned above and the prolonged duration of the pandemic, compliance fatigue has become increasingly prevalent among individuals, leading to the growing desire to restore pre-pandemic norms.

Correspondingly, in late January 2022, the governments of France [84] and Canada [85] pledged to begin coexisting with the pandemic, Italy [86] stepped up its rollout of vaccinations and vaccine passports, and Israel [87] ended a long-running state of emergency due to the pandemic. Living with COVID-19 requires flexibility in policy implementation to protect societies, economies, and individuals’ mental health while containing the spread of the disease.

This study also presented dynamic epidemic time series forecasting tools to analyze COVID-19. Our model can accurately predict and interpret external variables. Overall, all models' performances are well. We observed that certain exogenous regressors did not demonstrate better performance in comparison to the univariate model. This result is consistent with the findings of Nassiri et al. [44] who suggest accounting other variables may enhance the predictive capability.

Our models have many advantages and can help government agencies and public health professionals to fight COVID-19. First, they are easy to understand, intuitive, and straightforward. Second, their real-time nature is more suitable for the prevention of epidemics, which are characterized by constant dynamic changes. The models support decision-making about policies as new variants or outbreaks emerge with easy scenario simulation.

The ARIMA model has been widely utilized and demonstrated remarkable success in forecasting the progression of infectious diseases [88, 89]. Building upon this, the regARIMA model, which we have proposed by integrating exogenous variables, not only exhibits improved accuracy but also holds great promise for further applications in the relevant fields. Moreover, despite the number of countries included in our study being limited due to the considerable time and effort required for data access and processing, we made diligent efforts to comprehensively account for diversity in geography, culture, and COVID policy. Consequently, we are confident that our findings possess significant potential for generalizability and offer valuable insights, particularly within high socio-economic settings.

This study has some limitations. Given that the data on protective behaviours relied on self-report measures, it is conceivable that there may be accuracy biases stemming from social desirability or imperfect memory. Moreover, YouGov claims that individuals experiencing severe symptoms, those who have been hospitalized, and other challenging-to-reach groups may be inadequately represented within the sample. Furthermore, it is important to acknowledge that the prediction provided by this model is specifically tailored to SARS-CoV-2 variants. Given the constantly evolving nature of respiratory viruses and their distinct characteristics, it is crucial to involve incorporating diverse exogenous variables or exploring alternative modelling methodologies accordingly to better encapsulate the intricate dynamics and complexities inherent in different circumstances for giving more reliable predictions. In addition, the algorithm of fully vaccination is constrained by the types of data it has access to, which may not necessarily fully represent real-world conditions accurately.

## Conclusions

Our real-time model incorporating human behavior (wearing masks and avoiding going outside) and vaccination (fully vaccinated and given booster doses) variables performed well in two periods of the COVID-19 pandemic (Alpha & Delta and Omicron). Furthermore, by leveraging the model, our findings assisted in identifying and quantifying the significant determinants, such as human behaviour and vaccination, that play a crucial role in containing pandemics. Our development model can provide a reference for public health departments to formulate policies to deal with new variants of COVID-19 or emerging infectious diseases.

### Supplementary Information

**Additional file 1: S1 Fig.** Effects of full vaccination on the (A) case growth rate, (B) number of ICU patients with COVID-19 per million, and (C) number of deaths due to COVID-19 per million in the Omicron period. *p < 0.05, **p < 0.01, ***p < 0.001. **S2 Fig.** The Alpha & Delta (blue shaded area) and the Omicron periods (pink shaded area) based on the vaccine coverage for (a) full vaccination and (b) booster doses in Canada (red line), France (green line), Israel (blue line), and Italy (purple line). **S1 Appendix.** ARIMA model. **S2 Appendix.** case growth rate and first differences. **S1 Table.** Coefficient estimates of all models with/without adjusting for vaccine coverage in the Alpha & Delta period. **S2 Table.** Coefficient estimates of all models with/without adjusting by vaccine coverage in the Omicron period. (split at a 70:30 ratio).  **S3 Table.** Coefficient estimates of all models with/without adjusting for vaccine coverage in the Alpha & Delta period (split at an 80:20 ratio). **S4 Table.** Coefficient estimates of all models with/without adjusting for vaccine coverage in the Alpha & Delta period (split at a 90:10 ratio). **S5 Table.** Coefficient estimates of all models with/without adjusting by vaccine coverage in the Omicron period. (split at an 80:20 ratio). **S6 Table.** Coefficient estimates of all models with/without adjusting by vaccine coverage in the Omicron period (split at a 90:10 ratio).

## Data Availability

All data generated or analyzed during this study are included in this published article and its supplementary information files. These raw data are openly available for public access at YouGov data [https://github.com/YouGov-Data/covid-19-tracker/tree/fe0142dbf23b30755df537bacce5c6d687c52fdd] and Our World in Data [https://github.com/owid/covid-19-data/tree/master/public/data]. R code is available at the GitHub repository [https://github.com/ChiehCheng/ARIMA_forecast].

## Abbreviations

ARIMA: Automatic regressive integrated moving average
COVID-19: 2019 coronavirus disease
SARS-CoV-2: Severe acute respiratory syndrome coronavirus 2
regARIMA: Regression model with ARIMA errors
ICU: Intensive care unit
RMSE: Root mean square error
AICc: Corrected Akaike information criterion
AR: Autoregressive
I: Integration
MA: Moving average
ACF: Autocorrelation function
PACF: Partial autocorrelation function
ARIMAX: ARIMA models with explanatory variables
