# Supplementary material for: Real-time forecasting of COVID-19 spread according to protective behavior and vaccination: autoregressive integrated moving average models
Source: BMC Public Health. 2023 Aug 8;23:1500. doi: 10.1186/s12889-023-16419-8 (PMC10408098; doi:10.1186/s12889-023-16419-8)
Supplement: Supplementary file 1 — Additional file 1: S1 Fig. Effects of full vaccination on the (A) case growth rate, (B) number of ICU patients with COVID-19 per million, and (C) number of deaths due to COVID-19 per million in the Omicron period. *p < 0.05, **p < 0.01, ***p < 0.001. S2 Fig. The Alpha & Delta (blue shaded area) and the Omicron periods (pink shaded area) based on the vaccine coverage for (a) full vaccination and (b) booster doses in Canada (red line), France (green line), Israel (blue line), and Italy (purple line). S1 Appendix. ARIMA model. S2 Appendix. case growth rate and first differences. S1 Table. Coefficient estimates of all models with/without adjusting for vaccine coverage in the Alpha & Delta period. S2 Table. Coefficient estimates of all models with/without adjusting by vaccine coverage in the Omicron period. (split at a 70:30 ratio). S3 Table. Coefficient estimates of all models with/without adjusting for vaccine coverage in the Alpha & Delta period (split at an 80:20 ratio). S4 Table. Coefficient estimates of all models with/without adjusting for vaccine coverage in the Alpha & Delta period (split at a 90:10 ratio). S5 Table. Coefficient estimates of all models with/without adjusting by vaccine coverage in the Omicron period. (split at an 80:20 ratio). S6 Table. Coefficient estimates of all models with/without adjusting by vaccine coverage in the Omicron period (split at a 90:10 ratio). [file 12889_2023_16419_MOESM1_ESM.docx]

**Additional files**

S1 Fig. Effects of full vaccination on the (A) case growth rate, (B) number of ICU patients with COVID-19 per million, and (C) number of deaths due to COVID-19 per million in the Omicron period. **p* < 0.05, ***p* < 0.01, ****p* < 0.001.


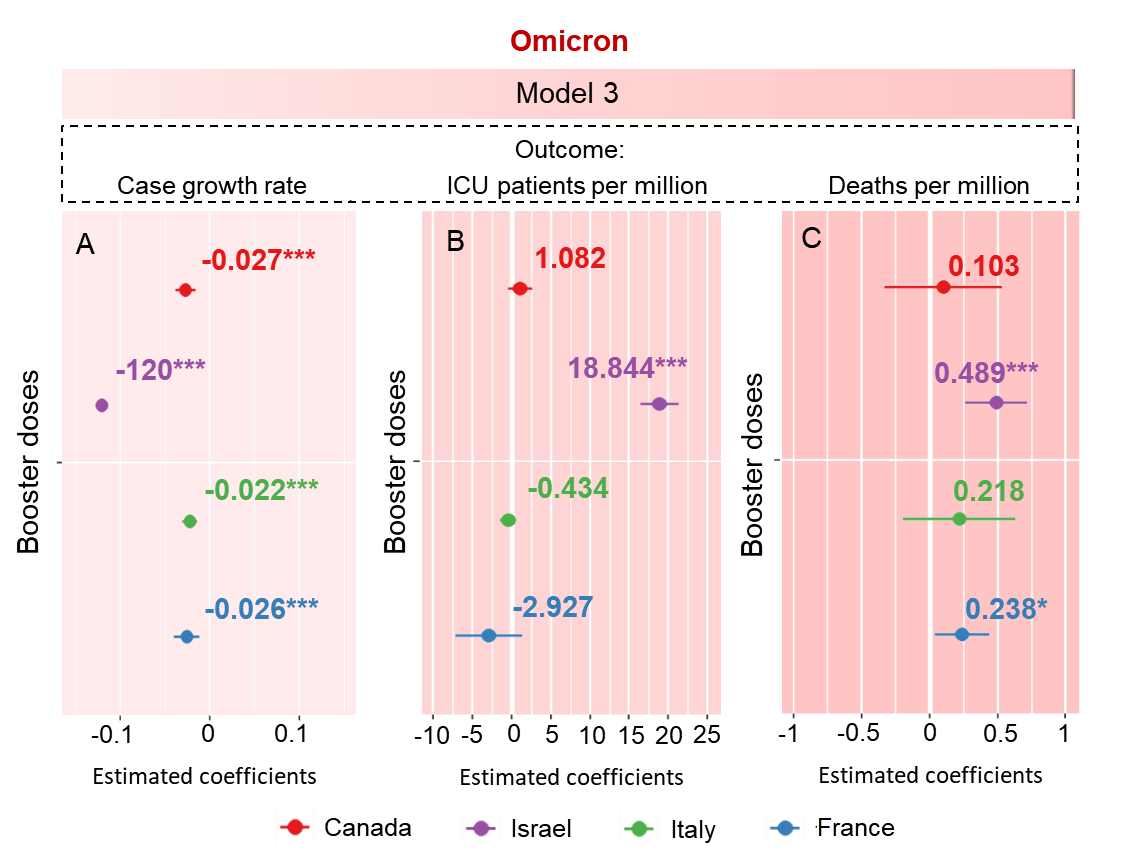


S2 Fig. The Alpha & Delta (blue shaded area) and the Omicron periods (pink shaded area) based on the vaccine coverage for (a) full vaccination and (b) booster doses in Canada (red line), France (green line), Israel (blue line), and Italy (purple line).


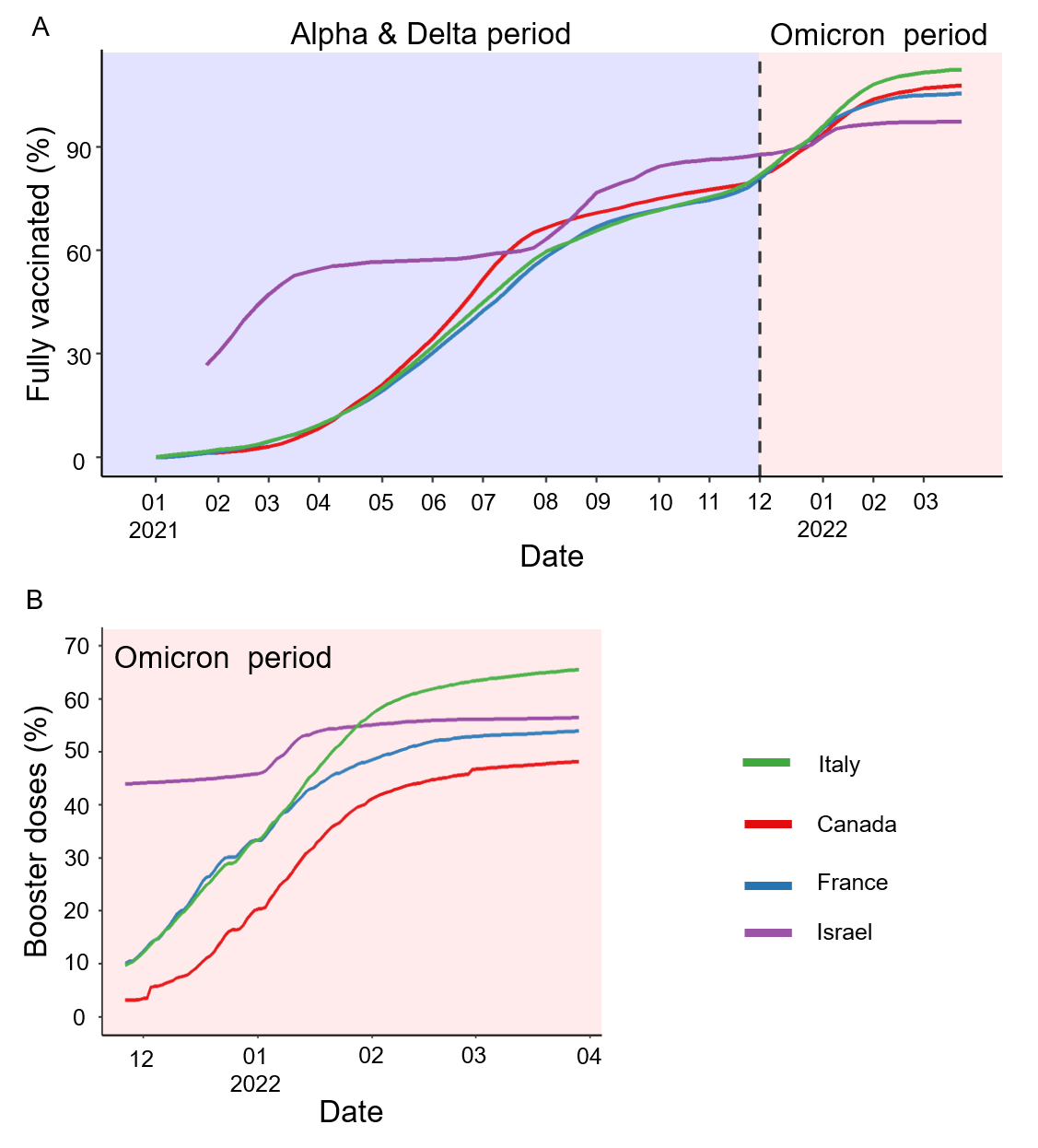


**S1 Appendix: ARIMA model**

The ARIMA model is a generalization of Auto Regressive Moving Average (ARMA). The AR part of ARIMA indicates that the variable is being regressed against itself. The AR can be written as:

$$y_{t}=\sum_{i=1}^{p} \varphi_{i}y_{t-i}+C+\varepsilon_{t}$$

Where $p$ is the order and $\varphi$ is the parameter of AR; $C$ is a constant; and $\varepsilon_{t}$ is white noise for the $t$ time. The MA part of ARIMA indicates that the past forecast errors. The MA can be written as:

$$y_{t}=\sum_{j=1}^{q} \theta_{i}\varepsilon_{t-i}+\varepsilon_{t}$$

Where $p$ is the order and $\varphi$ is the parameter of AR. For accurate forecasting, the time series is stationary by differencing. Overall, ARIMA model is expressed as ARIMA (*p, d, q*), where *p* and *q* as stated above and *d* is degree of differences. The full model is as follows:

$$y_{t} =C+\sum_{i=1}^{p} \varphi_{i}y_{t-i}+\sum_{j=1}^{q} \theta_{i}\varepsilon_{t-i}+\varepsilon_{t}$$

**S2 Appendix: case growth rate and first differences**

We observe that the time series' growth rate can be approximated by the first difference between its log levels, which is adapted from macroeconomics. This explains the reason for almost d=0 in S1 and S2 Table. In other words, the case growth rate is already in the first difference.

We show the derivation using a Taylor expansion as follows:

$$\Delta lnC_{t}=lnC_{t}- lnC_{t-1}$$

$$=ln\left( \frac{C_{t}}{C_{t-1}} \right)$$

$$=ln\left( 1+\frac{C_{t}-C_{t-1}}{C_{t-1}} \right)$$

$$\approx\frac{C_{t}-C_{t-1}}{C_{t-1}}$$

where $C_{t}$ is the number of cases in week *t*; $C_{t-1}$ is the number of cases in the last week.

S1 Table. Coefficient estimates of all models with/without adjusting for vaccine coverage in the Alpha & Delta period

| Outcome: case growth rate | | | | | | |
| --- | --- | --- | --- | --- | --- | --- |
| country | Model 1 | Model 2 | Model 3 | Model 4 | Model 5 | Model 6 |
| **Canada** | | | | | | |
| Parameters (p, d, q) | (1,0,0) | (1,0,0) | (1,0,0) | (1,0,0) | (1,0,0) | (1,0,0) |
| Mask wearing | -0.807* | - | - | -0.701 | - | - |
| Avoiding going out | - | -0.542* | - | - | -0.075 | - |
| fully vaccination | - | - | 0.475 | 0.437 | 0.745 | - |
| **Israel** | | | | | | |
| Parameters (p, d, q) | (1,0,0) | (1,0,0) | (0,1,0) | (1,0,0) | (1,0,0) | (0,1,0) |
| Mask wearing | -1.107*** | - | - | -1.101*** | - | - |
| Avoiding going out | - | -0.032 | - | - | -0.110 | - |
| fully vaccination | - | - | -0.273 | 0.055 | 0.340 | - |
| **Italy** | | | | | | |
| Parameters (p, d, q) | (0,0,2) | (2,0,0) | (2,0,0) | (0,0,2) | (2,0,0) | (2,0,0) |
| Mask wearing | -1.263*** | - | - | -1.268*** | - | - |
| Avoiding going out | - | -0.023 | - | - | -0.049 | - |
| fully vaccination | - | - | 0.106 | -0.015 | 0.368 | - |
| **France** | | | | | | |
| Parameters (p, d, q) | (1,0,1) | (2,0,0) | (1,0,1) | (1,0,1) | (2,0,0) | (1,0,1) |
| Mask wearing | -0.003 | - | - | -0.020 | - | - |
| Avoiding going out | - | -0.207 | - | - | -0.045 | - |
| fully vaccination | - | . | 0.153 | 0.337 | 0.467 | - |

**p* < 0.05, ***p* < 0.01, ****p* < 0.001.

S2 Table. Coefficient estimates of all models with/without adjusting by vaccine coverage in the Omicron period. (split at a 70:30 ratio)

| Outcome: case growth rate | | | | | | |
| --- | --- | --- | --- | --- | --- | --- |
| country | Model 1 | Model 2 | Model 3 | Model 4 | Model 5 | Model 6 |
| **Canada** | | | | | | |
| Parameters (p, d, q) | (1,0,0) | (0,1,0) | (0,0,0) | (0,0,0) | (0,0,0) | (0,1,0) |
| Mask wearing | 0.055 | - | - | 0.195*** | - | - |
| Avoiding going out | - | 0.300 | - | - | 0.284*** | - |
| booster doses | - | - | -0.027*** | -0.026*** | -0.026*** | - |
| **Israel** | | | | | | |
| Parameters (p, d, q) | (0,1,0) | (1,0,0) | (0,0,0) | (0,0,0) | (0,0,0) | (0,1,0) |
| Mask wearing | -2.144 | - | - | 1.808*** | - | - |
| Avoiding going out | - | -0.086 | - | - | 1.682* | - |
| booster doses | - | - | -0.120*** | -0.127*** | -0.184*** | - |
| **Italy** | | | | | | |
| Parameters (p, d, q) | (0,0,0) | (0,1,0) | (0,0,0) | (0,0,0) | (0,0,0) | (0,1,0) |
| Mask wearing | -7.653*** | - | - | -6.087** | - | - |
| Avoiding going out | - | -0.793 | - | - | -0.809* | - |
| booster doses | - | - | -0.022*** | -0.006 | -0.017*** | - |
| **France** | | | | | | |
| Parameters (p, d, q) | (0,1,0) | (0,1,0) | (0,0,0) | (0,0,0) | (0,0,0) | (0,1,0) |
| Mask wearing | 0.100 | - | - | 0.268*** | - | - |
| Avoiding going out | - | -0.319 | - | - | 0.477** | - |
| booster doses | - | - | -0.026*** | -0.025*** | -0.0246 | - |

**p* < 0.05, ***p* < 0.01, ****p* < 0.001.

S3 Table. Coefficient estimates of all models with/without adjusting for vaccine coverage in the Alpha & Delta period (split at an 80:20 ratio)

| Outcome: case growth rate | | | | | | |
| --- | --- | --- | --- | --- | --- | --- |
| country | Model 1 | Model 2 | Model 3 | Model 4 | Model 5 | Model 6 |
| **Canada** | | | | | | |
| Parameters (p, d, q) | (1,0,0) | (1,0,0) | (1,0,0) | (1,0,0) | (1,0,0) | (1,0,0) |
| Mask wearing | -0.717* | - | - | -0.671* | - | - |
| Avoiding going out | - | -0.020 | - | - | -0.056 | - |
| fully vaccination | - | - | 0.192 | 0.123 | 0.399 | - |
| RMSE | 0.1316 | 0.1403 | 0.1398 | 0.1314 | 0.1376 | 0.1407 |
| **Israel** | | | | | | |
| Parameters (p, d, q) | (1,0,0) | (1,0,0) | (1,0,0) | (1,0,0) | (1,0,0) | (0,1,0) |
| Mask wearing | -1.095*** | - | - | -1.084*** | -0.097 | - |
| Avoiding going out | - | -0.040 | - | - | - | - |
| fully vaccination | - | - | 0.008 | 0.162 | 0.220 |  |
| RMSE | 0.2514 | 0.3215 | 0.3219 | 0.2513 | 0.3210 | 0.3397 |
| **Italy** | | | | | | |
| Parameters (p, d, q) | (1,0,2) | (2,0,0) | (2,0,0) | (1,0,2) | (2,0,0) | (2,0,0) |
| Mask wearing | -0.951** | - | - | -1.034** | - | - |
| Avoiding going out | - | -0.026 | - | - | -0.038 | - |
| fully vaccination | - | - | -0.026 | -0.225 | 0.130 |  |
| RMSE | 0.1332 | 0.1439 | 0.1451 | 0.1322 | 0.1435 | 0.1451 |
| **France** | | | | | | |
| Parameters (p, d, q) | (1,0,1) | (1,0,1) | (1,0,1) | (1,0,1) | (1,0,1) | (1,0,1) |
| Mask wearing | -0.014 | - | - | -0.009 | - | - |
| Avoiding going out | - | -0.022 | - | - | -0.016 | - |
| fully vaccination | - | - | -0.144 | -0.071 | -0.084 | - |
| RMSE | 0.1550 | 0.1552 | 0.1552 | 0.1550 | 0.1550 | 0.1557 |

RMSE: root mean square error

**p* < 0.05, ***p* < 0.01, ****p* < 0.001.

S4 Table. Coefficient estimates of all models with/without adjusting for vaccine coverage in the Alpha & Delta period (split at a 90:10 ratio)

| Outcome: case growth rate | | | | | | |
| --- | --- | --- | --- | --- | --- | --- |
| country | Model 1 | Model 2 | Model 3 | Model 4 | Model 5 | Model 6 |
| **Canada** | | | | | | |
| Parameters (p, d, q) | (1,0,0) | (1,0,0) | (1,0,0) | (1,0,0) | (1,0,0) | (1,0,0) |
| Mask wearing | -0.833*** | - | - | -0.844** | - | - |
| Avoiding going out | - | -0.027 | - | - | -0.051 | - |
| fully vaccination | - | - | 0.036 | -0.046 | 0.221 | - |
| RMSE | 0.1300 | 0.1461 | 0.1469 | 0.1300 | 0.1450 | 0.1469 |
| **Israel** | | | | | | |
| Parameters (p, d, q) | (1,0,0) | (1,0,0) | (1,0,0) | (1,0,0) | (1,0,0) | (1,0,0) |
| Mask wearing | -1.088*** | - | - | -1.132*** | - | - |
| Avoiding going out | - | -0.069 | - | - | -0.039 | - |
| fully vaccination | - | - | -0.194 | -0.506 | -0.118 | - |
| RMSE | 0.2454 | 0.3077 | 0.3075 | 0.2433 | 0.3074 | 0.3088 |
| **Italy** | | | | | | |
| Parameters (p, d, q) | (0,0,3) | (2,0,0) | (2,0,0) | (0,0,3) | (2,0,0) | (2,0,0) |
| Mask wearing | -0.908*** | - | - | -0.936*** | - | - |
| Avoiding going out | - | -0.020 | - | - | -0.041 | - |
| fully vaccination | - | - | 0.045 | -0.098 | 0.183 | - |
| RMSE | 0.1277 | 0.1443 | 0.1449 | 0.1277 | 0.1431 | 0.1450 |
| **France** | | | | | | |
| Parameters (p, d, q) | (1,0,1) | (1,0,1) | (1,0,1) | (1,0,1) | (1,0,1) | (1,0,1) |
| Mask wearing | -0.010 | - | - | -0.011 | - | - |
| Avoiding going out | - | -0.019 | - | - | -0.019 | - |
| fully vaccination | - | - | -0.064 | 0.005 | 0.001 | - |
| RMSE | 0.1480 | 0.1479 | 0.1482 | 0.1480 | 0.1479 | 0.1483 |

RMSE: root mean square error

**p* < 0.05, ***p* < 0.01, ****p* < 0.001.

S5 Table. Coefficient estimates of all models with/without adjusting by vaccine coverage in the Omicron period. (split at an 80:20 ratio)

| Outcome: case growth rate | | | | | | |
| --- | --- | --- | --- | --- | --- | --- |
| country | Model 1 | Model 2 | Model 3 | Model 4 | Model 5 | Model 6 |
| **Canada** | | | | | | |
| Parameters (p, d, q) | (1,0,0) | (0,1,0) | (0,0,1) | (0,0,0) | (0,0,0) | (0,1,0) |
| Mask wearing | 0.061 | - | - | 0.179*** | - | - |
| Avoiding going out | - | 0.209 | - | - | -0.819* | - |
| booster doses | - | - | -0.022** | -0.022*** | -0.026*** | - |
| AICc | 11.07 | 7.71 | 12.3 | 12.28 | 13.84 | 5.05 |
| **Israel** | | | | | | |
| Parameters (p, d, q) | (0,1,0) | (0,1,0) | (0,0,0) | (0,0,0) | (0,0,0) | (0,1,0) |
| Mask wearing | -2.151 | - | - | 1.746*** | - | - |
| Avoiding going out | - | -0.466 | - | - | 1.383 | - |
| booster doses | - | - | -0.120*** | -0.122*** | -0.166*** | - |
| AICc | 14.82 | 16.5 | 17.87 | 18.2 | 19.41 | 13.76 |
| **Italy** | | | | | | |
| Parameters (p, d, q) | (0,1,0) | (0,1,0) | (0,0,0) | (0,0,0) | (0,0,0) | (0,1,0) |
| Mask wearing | -2.531 | - | - | -3.240** | - | - |
| Avoiding going out | - | -0.800 | - | - | -0.848** | - |
| booster doses | - | - | -0.020*** | -0.014*** | -0.016*** | - |
| AICc | 5.06 | 4.76 | 7.86 | 7.29 | 7.2 | 3.93 |
| **France** | | | | | | |
| Parameters (p, d, q) | (0,1,0) | (0,1,0) | (0,0,1) | (0,0,0) | (0,0,0) | (0,1,0) |
| Mask wearing | 0.104 | - | - | 0.251*** | - | - |
| Avoiding going out | - | -0.381 | - | - | 0.419** | - |
| booster doses | - | - | -0.019* | -0.023*** | -0.021** | - |
| AICc | 9.53 | 8.95 | 12.47 | 12.35 | 13.87 | 6.54 |

AICc: corrected Akaike information criterion

**p* < 0.05, ***p* < 0.01, ****p* < 0.001.

S6 Table. Coefficient estimates of all models with/without adjusting by vaccine coverage in the Omicron period (split at a 90:10 ratio)

| Outcome: case growth rate | | | | | | |
| --- | --- | --- | --- | --- | --- | --- |
| country | Model 1 | Model 2 | Model 3 | Model 4 | Model 5 | Model 6 |
| **Canada** | | | | | | |
| Parameters (p, d, q) | (1,0,0) | (0,1,0) | (1,0,0) | (0,0,0) | (0,0,0) | (0,1,0) |
| Mask wearing | 0.046 | - | - | 0.165 *** | - | - |
| Avoiding going out | - | 0.123 | - | - | -0.865* | - |
| booster doses | - | - | -0.021** | -0.020 | -0.024 | - |
| AICc | 9.40 | 6.54 | 8.99 | 11.49 | 11.09 | 3.88 |
| **Israel** | | | | | | |
| Parameters (p, d, q) | (0,1,0) | (0,1,0) | (0,0,0) | (0,0,0) | (0,0,0) | (0,1,0) |
| Mask wearing | -2.169 | - | - | 1.417*** | - | - |
| Avoiding going out | - | -0.500 | - | - | 0.004* | - |
| booster doses | - | - | -0.104*** | -0.097 | -0.104 | - |
| AICc | 15.42 | 17.22 | 20.19 | 22.4 | 24.23 | 14.89 |
| **Italy** | | | | | | |
| Parameters (p, d, q) | (0,1,0) | (0,1,0) | (0,0,1) | (0,0,0) | (0,0,0) | (0,1,0) |
| Mask wearing | -2.522 | - | - | -4.349*** | - | - |
| Avoiding going out | - | -0.752 | - | - | -1.219*** | - |
| booster doses | - | - | -0.013* | -0.011 | -0.011 | - |
| AICc | 5.52 | 5.32 | 11.46 | 4.58 | 7.57 | 4.57 |
| **France** | | | | | | |
| Parameters (p, d, q) | (0,1,0) | (0,1,0) | (0,0,1) | (0,0,0) | (0,0,0) | (0,1,0) |
| Mask wearing | 0.097 | - | - | 0.197* | - | - |
| Avoiding going out | - | -0.386 | - | - | -0.857 | - |
| booster doses | - | - | -0.012 | -0.016 | -0.021 | - |
| AICc | 9.15 | 8.36 | 11.9 | 16.42 | 17.9 | 6.38 |

AICc: corrected Akaike information criterion

**p* < 0.05, ***p* < 0.01, ****p* < 0.001.
